# A cellular screen identifies ponatinib and pazopanib as inhibitors of necroptosis

**DOI:** 10.1038/cddis.2015.130

**Published:** 2015-05-21

**Authors:** A Fauster, M Rebsamen, K V M Huber, J W Bigenzahn, A Stukalov, C-H Lardeau, S Scorzoni, M Bruckner, M Gridling, K Parapatics, J Colinge, K L Bennett, S Kubicek, S Krautwald, A Linkermann, G Superti-Furga

**Affiliations:** 1 CeMM Research Center for Molecular Medicine of the Austrian Academy of Sciences, Vienna, Austria; 2 Division of Nephrology and Hypertension, Christian-Albrechts-University, Kiel 24105 Germany

## Abstract

Necroptosis is a form of regulated necrotic cell death mediated by receptor-interacting serine/threonine-protein kinase 1 (RIPK1) and RIPK3. Necroptotic cell death contributes to the pathophysiology of several disorders involving tissue damage, including myocardial infarction, stroke and ischemia-reperfusion injury. However, no inhibitors of necroptosis are currently in clinical use. Here we performed a phenotypic screen for small-molecule inhibitors of tumor necrosis factor-alpha (TNF*-α*)-induced necroptosis in Fas-associated protein with death domain (FADD)-deficient Jurkat cells using a representative panel of Food and Drug Administration (FDA)-approved drugs. We identified two anti-cancer agents, ponatinib and pazopanib, as submicromolar inhibitors of necroptosis. Both compounds inhibited necroptotic cell death induced by various cell death receptor ligands in human cells, while not protecting from apoptosis. Ponatinib and pazopanib abrogated phosphorylation of mixed lineage kinase domain-like protein (MLKL) upon TNF-*α*-induced necroptosis, indicating that both agents target a component upstream of MLKL. An unbiased chemical proteomic approach determined the cellular target spectrum of ponatinib, revealing key members of the necroptosis signaling pathway. We validated RIPK1, RIPK3 and transforming growth factor-*β*-activated kinase 1 (TAK1) as novel, direct targets of ponatinib by using competitive binding, cellular thermal shift and recombinant kinase assays. Ponatinib inhibited both RIPK1 and RIPK3, while pazopanib preferentially targeted RIPK1. The identification of the FDA-approved drugs ponatinib and pazopanib as cellular inhibitors of necroptosis highlights them as potentially interesting for the treatment of pathologies caused or aggravated by necroptotic cell death.

Programmed cell death has a crucial role in a variety of biological processes ranging from normal tissue development to diverse pathological conditions.^1, 2^ Necroptosis is a form of regulated cell death that has been shown to occur during pathogen infection or sterile injury-induced inflammation in conditions where apoptosis signaling is compromised.^3, 4, 5, 6^ Given that many viruses have developed strategies to circumvent apoptotic cell death, necroptosis constitutes an important, pro-inflammatory back-up mechanism that limits viral spread *in vivo*.^7, 8, 9^ In contrast, in the context of sterile inflammation, necroptotic cell death contributes to disease pathology, outlining potential benefits of therapeutic intervention.^10^ Necroptosis can be initiated by death receptors of the tumor necrosis factor (TNF) superfamily,^11^ Toll-like receptor 3 (TLR3),^12^ TLR4,^13^ DNA-dependent activator of IFN-regulatory factors^14^ or interferon receptors.^15^ Downstream signaling is subsequently conveyed via RIPK1^16^ or TIR-domain-containing adapter-inducing interferon-*β*,^8, 17^ and converges on RIPK3-mediated^13, 18, 19, 20^ activation of MLKL.^21^ Phosphorylated MLKL triggers membrane rupture,^22, 23, 24, 25, 26^ releasing pro-inflammatory cellular contents to the extracellular space.^27^ Studies using the RIPK1 inhibitor necrostatin-1 (Nec-1) ^28^ or RIPK3-deficient mice have established a role for necroptosis in the pathophysiology of pancreatitis,^19^ artherosclerosis,^29^ retinal cell death,^30^ ischemic organ damage and ischemia-reperfusion injury in both the kidney^31^ and the heart.^32^ Moreover, allografts from RIPK3-deficient mice are better protected from rejection, suggesting necroptosis inhibition as a therapeutic option to improve transplant outcome.^33^ Besides Nec-1, several tool compounds inhibiting different pathway members have been described,^12, 16, 21, 34, 35^ however, no inhibitors of necroptosis are available for clinical use so far.^2, 10^ In this study we screened a library of FDA approved drugs for the precise purpose of identifying already existing and generally safe chemical agents that could be used as necroptosis inhibitors. We identified the two structurally distinct kinase inhibitors pazopanib and ponatinib as potent blockers of necroptosis targeting the key enzymes RIPK1/3.

## Results

### A drug screen in FADD-deficient Jurkat cells identifies ponatinib and pazopanib as necroptosis inhibitors

**Figure 1:**
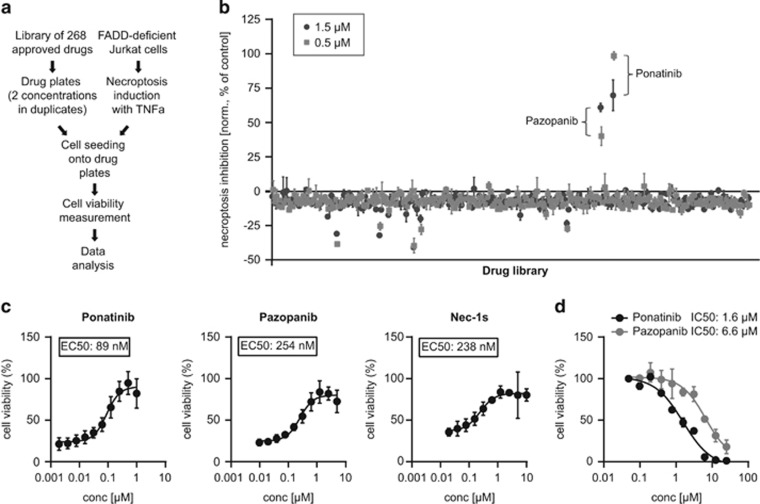
A cell-based drug screen identifies ponatinib and pazopanib as necroptosis inhibitors. (**a**) Schematic overview of the drug screen workflow. (**b**) Normalized necroptosis inhibition values depicted as percentage of control (DMSO=0, Nec-1 [10 *μ*M]=100) for drugs tested on FADD-deficient Jurkat cells treated overnight with 10 ng/ml TNF*-α*. Values represent mean value±S.D. for 268 drugs assayed at 1.5 (dark gray) or 0.5 *μ*M (light gray) in duplicates, respectively. (**c**) FADD-deficient Jurkat cells were treated overnight with 10 ng/ml TNF*-α* and ponatinib or pazopanib as indicated. Data were normalized to untreated control cells and represent mean value±S.D. of four independent experiments performed in triplicates. (**d**) FADD-deficient Jurkat cells were treated for 24 h with ponatinib or pazopanib as indicated. Data represent mean value±S.D. of two independent experiments performed in triplicates and normalized to untreated control. Cell viability was assessed using a luminescence-based readout for ATP (CellTiter Glo) throughout

To identify novel necroptosis inhibitors for potential clinical use, we screened a panel of 268 FDA-approved drugs with diverse mechanisms of action for their ability to block TNF-*α*-induced necroptosis in FADD deficient human Jurkat T-cells^36, 37^ (Figure 1a). We confirmed the validity of our experimental setup using the RIPK1 inhibitor Nec-1^28, 37^ (Supplementary Figure 1a). To selectively identify inhibitors effective at low concentrations, the compounds were assayed at 0.5 and 1.5 *μ*M. Among the drugs investigated, the protein kinase inhibitors ponatinib and pazopanib robustly rescued cell viability at both concentrations (Figure 1b). Ponatinib (AP24534) is an oral multi-targeted tyrosine kinase inhibitor developed for treatment of chronic myeloid leukemia and Philadelphia chromosome-positive acute lymphoblastic leukemia.^38, 39^ This BCR-ABL inhibitor is used as second-line treatment for patients who have acquired resistance to standard therapy. Pazopanib is an oral receptor tyrosine kinase inhibitor approved for treatment of patients with advanced renal cell carcinoma and soft tissue sarcoma^40, 41^ targeting vascular endothelial growth factor receptor (VEGFR)-1, -2, -3, platelet-derived growth factor receptor *β* (PDGFR*β*) and c-Kit.^42^ We confirmed the screening results (Supplementary Figures 1b–e) and performed dose–response curves to quantitatively assess the inhibitory potency of the two drugs. Pazopanib and ponatinib blocked necroptosis with comparable or higher efficiency than Nec-1 (data not shown) and its improved analog Nec-1s.^43^ The half maximal effective concentration (EC_50_) for inhibiting necroptosis in this setting was measured to be 89 nM for ponatinib, 254 nM for pazopanib and 238 nM for Nec-1s (Figure 1c). Drug toxicity was assessed by determining the half maximal inhibitory concentration (IC_50_) (Figure 1d and Supplementary Figure 1f), which was 1.6 *μ*M for ponatinib and 6.6 *μ*M for pazopanib, highlighting the window of opportunity for necroptosis inhibition.

### Ponatinib and pazopanib specifically block necroptotic but not apoptotic cell death triggered by various death receptor ligands in human cells

**Figure 2:**
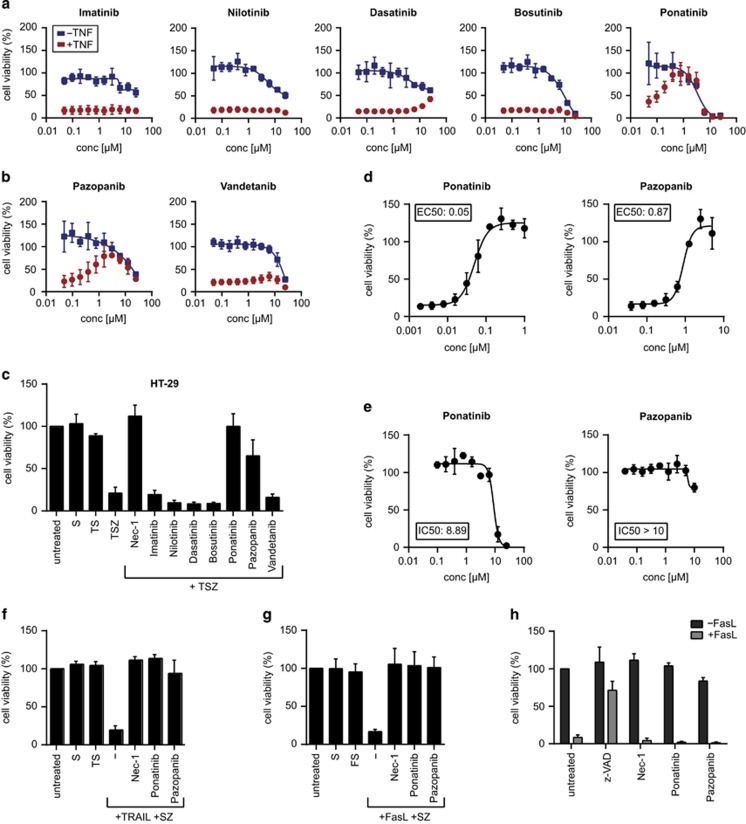
Ponatinib and pazopanib efficiently and specifically block necroptosis. (**a** and **b**) Cell viability was determined in FADD-deficient Jurkat cells treated overnight with (red circles) or without (blue rectangles) 10 ng/ml TNF*-α* and drugs as indicated. (**c**) Cell viability was assessed in HT-29 cells treated overnight with 20 ng/ml TNF-*α* (T), 500 nM Smac mimetic (S), 20 *μ*M caspase inhibitor z-VAD (Z) and the compounds indicated (Nec-1, 10 *μ*M; all others, 1 *μ*M). (**d**) HT-29 cells were treated with TSZ and ponatinib or pazopanib as indicated. (**e**) HT-29 cells were treated with ponatinib or pazopanib at concentrations indicated for 24 h. (**f**) Cell viability was assessed in HT-29 cells treated overnight with 200 ng/ml TRAIL or (**g**) 200 ng/ml human FasL together with 500 nM Smac mimetic (S), 20 *μ*M z-VAD (Z) and either 10 *μ*M Nec-1, 0.5 *μ*M ponatinib or 5 *μ*M pazopanib. Data represent mean value±S.D. of two independent experiments performed in triplicates and normalized to untreated control. (**h**) Cell viability was determined in Jurkat E6.1 cells treated with 100 ng/ml human FasL and 10 *μ*M z-VAD, 10 *μ*M Nec-1, 0.5 *μ*M ponatinib or 5 *μ*M pazopanib for 24 h. Data represent mean value±S.D. of two independent experiments performed in triplicates and normalized to untreated control. Cell viability was assessed using a luminescence-based readout for ATP (CellTiter Glo) throughout

Ponatinib is one of the five BCR-ABL inhibitors currently approved for clinical use, the others being imatinib, nilotinib, dasatinib and bosutinib.^44^ The target spectra of the latter four have been analyzed previously and show extensive overlap.^45, 46^ To investigate whether the effect of ponatinib was caused by a target shared with the other BCR-ABL inhibitors, we assayed their potential to block necroptosis and their toxicity (Figure 2a). In contrast to the protection conferred by ponatinib treatment, none of the other drugs prevented necroptotic cell death. Dasatinib showed a modest effect but only at drug concentrations high enough to induce toxicity. These results suggest that ponatinib mediates its protective effect through one or multiple targets, which are not shared by the other BCR-ABL inhibitors. Similarly, necroptosis inhibition by pazopanib did not appear to be mediated through its main targets, as vandetanib, another VEGFR inhibitor,^47^ did not confer protection (Figure 2b). To examine the inhibitory effect of the two drugs in an additional cellular model of programmed necrosis, we treated human adenocarcinoma HT-29 cells with TNF*-α* in presence of the Smac mimetic birinapant,^48^ and the pan-caspase inhibitor z-VAD-FMK. Ponatinib and pazopanib rescued TNF-*α*/Smac mimetic/z-VAD-FMK (TSZ)-induced necroptotic cell death, whereas neither of the other four BCR-ABL inhibitors nor vandetanib did (Figure 2c and Supplementary Figure 2a). Ponatinib blocked necroptosis more efficiently in HT-29 cells (EC_50_: 50 nM; Figure 2d) and was less toxic (IC_50_: 8.9 *μ*M; Figure 2e) than in FADD-deficient Jurkat cells. Pazopanib blocked necroptosis less potently in HT-29 cells (EC_50_: 873 nM; Figure 2d) while also showing reduced toxicity (IC_50_>10 *μ*M; Figure 2e). The inhibitory effect of the two drugs in HT-29 cells was not confined to TNF-driven necroptosis as cell death induced by TNF-related apoptosis-inducing ligand (TRAIL) and Fas ligand (FasL) was similarly blocked by ponatinib and pazopanib (Figures 2f and g and Supplementary Figure 2b). In contrast, none of the drugs inhibited apoptotic cell death triggered by FasL (Figure 2h and Supplementary Figure 2c). Similar to the inhibitory effect observed in human cells, both ponatinib and pazopanib were also capable of blocking necroptotic cell death with comparable potency in murine 3T3-SA cells (Supplementary Figures 3a and b). In L929 cells, ponatinib conferred protection as well, yet the effect was partial and confined to a small dosage window around 100 nM, above which the combined treatment of ponatinib, TNF-*α* and z-VAD-FMK induced toxicity (Supplementary Figures 3c and d). Altogether these data demonstrate that ponatinib and pazopanib are potent inhibitors of necroptosis.

### Chemical proteomics identifies necroptosis pathway members as targets of ponatinib

**Figure 3:**
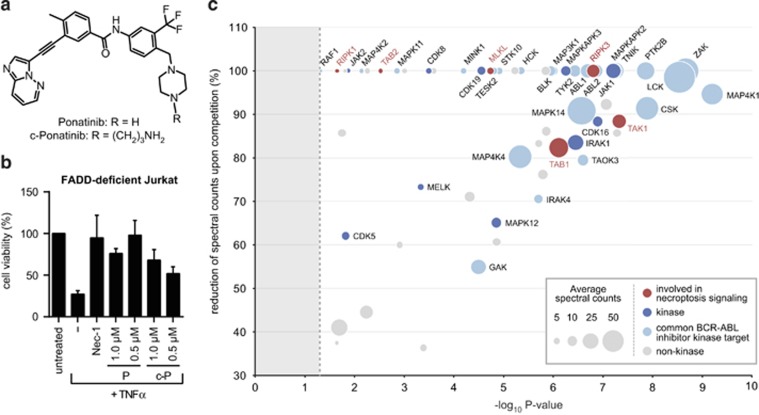
Chemical proteomics identifies necroptosis pathway members as targets of ponatinib. (**a**) Structure of ponatinib and the analog c-ponatinib used for affinity purification. (**b**) Cell viability was determined in FADD-deficient Jurkat cells treated overnight with 10 ng/ml TNF-*α*, 10 *μ*M Nec-1 and ponatinib (P) or c-ponatinib (c-P) as indicated. Cell viability was assessed using a luminescence-based readout for ATP (CellTiter Glo). Data represent mean value±S.D. of two independent experiments performed in triplicates and normalized to untreated control. (**c**) Proteins identified in mass-spectrometry-based affinity purification experiment with c-ponatinib. The *x*-axis of the bubble plot represents the statistical significance (*P*-value) of protein enrichment over the competition assay with free ponatinib estimated using the modified Decontaminator method (see Materials and Methods), and the *y*-axis the reduction in spectral counts (%) upon competition. Bubble size is proportional to the average spectral counts in non-competed condition. Dashed line indicates the *P*-value threshold (0.05)

To characterize the molecular mode of action by which the two drugs mediate necroptosis inhibition, we used a chemical proteomic strategy to identify the cellular targets of ponatinib,^49^ as pazopanib has been described to bind RIPK1.^50^ We designed an analog of ponatinib (c-ponatinib) that contained an *N*-aminopropyl linker (Figure 3a), providing the basis for coupling the compound to a bead matrix and thereby allowing subsequent affinity purification of drug-binding proteins. We confirmed that the modification did not interfere with the necroptosis inhibitory capacity of ponatinib. Indeed, c-Ponatinib blocked necroptosis with only a minor reduction in potency (Figure 3b). For drug pull-down experiments, we treated FADD-deficient Jurkat cells for two hours with TNF*-α* to induce necroptotic signaling complex formation followed by cell lysis. The lysates were incubated with the c-ponatinib drug matrix in presence or absence of an excess of competing free ponatinib.^51^ Analysis of the competed samples enabled discrimination of *bona fide* drug binders from contaminant proteins interacting with the bead matrix. Liquid chromatography tandem mass spectrometry (LCMS-MS) followed by bioinformatic analysis revealed a total of 38 kinases and 22 non-kinase proteins (Figure 3c and Supplementary Table 1) specific for ponatinib. The comparison with previously determined target profiles of other BCR-ABL inhibitors^45, 46^ revealed a large overlap in the target spectra. Twenty-three of the 38 identified kinases have been observed to interact with at least one of the other BCR-ABL inhibitors. Of note, among the proteins unique to the target spectrum of ponatinib, we found all the key components of the necroptosis machinery: RIPK1, RIPK3 and MLKL. In addition, we identified TAK1, TGF-beta-activated kinase 1 and MAP3K7-binding protein 1 (TAB1) and TAB2, key components of the TNF-signaling pathway, which have recently been proposed to also exert a regulatory function in necroptotic cell death.^52, 53^ The specificity of these necroptosis-relevant proteins for ponatinib was in accordance with the absence of necroptosis inhibition observed with the other clinical BCR-ABL inhibitors (Figure 2a). The chemical proteomic approach provided a target profile of ponatinib that comprised all crucial components of the necroptosis signaling pathway, perfectly in line with the inhibitory effect observed.

### Ponatinib blocks necroptosis by inhibiting RIPK1 and RIPK3 activity

**Figure 4:**
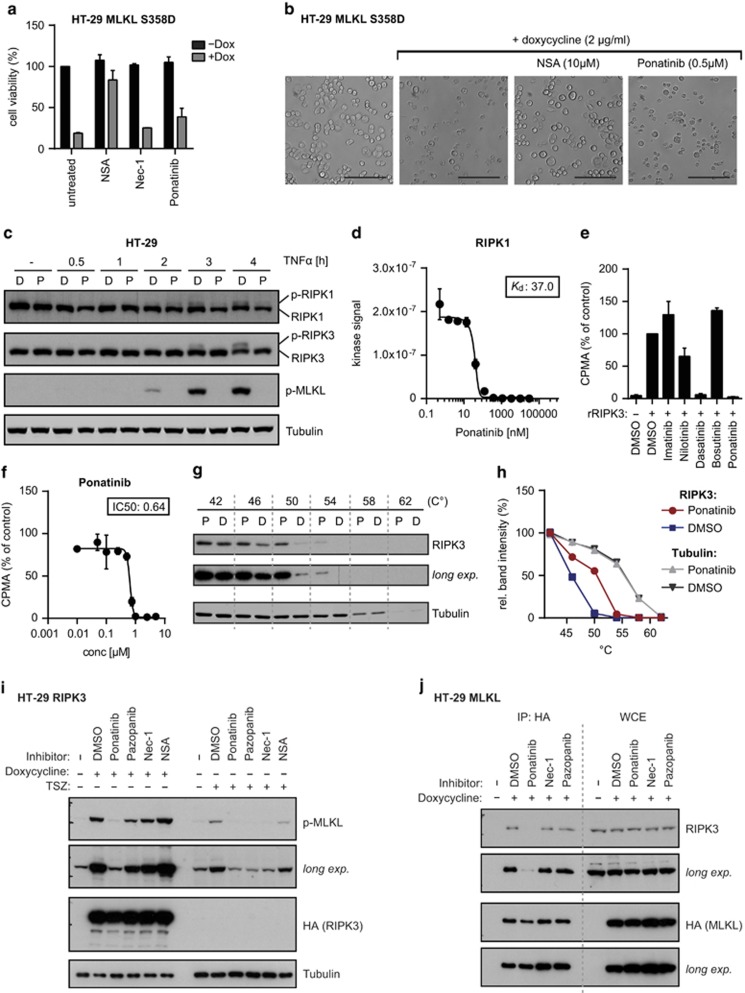
RIPK1 and RIPK3 are targets of ponatinib. (**a**) Cell viability was determined in HT-29 cells with doxycycline-inducible MLKL S358D expression treated overnight with 2 *μ*g/ml doxycycline and 10 *μ*M NSA, 10 *μ*M Nec-1 or 0.5 *μ*M ponatinib. Cell viability was assessed using a luminescence-based readout for ATP (CellTiter Glo). Data represent mean value±S.D. of three independent experiments performed in triplicates and normalized to untreated control. (**b**) Microscopy (brightfield, × 10) of HT-29 MLKL S358D cells induced with 2 *μ*g/ml doxycycline overnight and treated with the compounds as indicated. Scale bar, 100 *μ*m. (**c**) HT-29 cells were treated with 500 nM Smac mimetic, 20 *μ*M z-VAD, 0.5 *μ*M ponatinib (P) or DMSO (D) for a total of 4.5 h in the presence of TNF*-α* (10 ng/ml) for the time indicated. Cells were lysed and immunoblotted with the indicated antibodies. Data shown are representative of two independent experiments. (**d**) Direct binding assay for ponatinib and RIPK1. Data represent mean value±S.D. of two independent experiments. (**e**) *In vitro* kinase assay using recombinant RIPK3. Phosphorylation of MBP was monitored in presence of 10 *μ*M of the kinase inhibitors stated or (**f**) ponatinib as indicated. Data represent mean value±S.D. of two independent experiments normalized to DMSO control. (**g**) CETSA performed in FADD-deficient Jurkat cells treated with 500 nM ponatinib (P) or DMSO (D) control. Cells were lysed by three freeze–thaw cycles and immunoblotted with the antibodies indicated. Data shown are representative of two independent experiments. (**h**) Quantification of band intensity (ImageJ) of RIPK3 and Tubulin immunoblots shown in **g**. (**i**) HT-29 cells with doxycycline-inducible expression of HA-tagged RIPK3 were treated with 0.5 *μ*M ponatinib, 5 *μ*M pazopanib, 10 *μ*M Nec-1 or 10 *μ*m NSA and stimulated overnight with 1 *μ*g/ml doxycycline or for 2 h with 10 ng/ml TNF*-α*, 500 nM Smac mimetic and 20 *μ*M z-VAD. Cells were lysed and immunoblotted with the indicated antibodies. Data shown are representative of two independent experiments. (**j**) HT-29 cells with doxycycline-inducible expression of HA-tagged MLKL were treated overnight with 1 *μ*g/ml doxycycline in presence of 0.5 *μ*M ponatinib, 5 *μ*M pazopanib, 10 *μ*M Nec-1 or 10 *μ*M NSA. Cell lysates were subjected to immunoprecipitation and immunoprecipitates (IP) and whole cell extracts (WCE) were analyzed by immunoblotting with the indicated antibodies. Data shown are representative of two independent experiments

Given that RIPK1, RIPK3 and MLKL interact upon necroptosis induction,^21, 54^ we set out to assess which of them constitutes a direct target of ponatinib. To test the inhibitory effect of ponatinib on the pseudokinase MLKL, we generated HT-29 cells expressing a constitutively active MLKL S358D mutant upon doxycycline treatment (Supplementary Figure 4a). Induction of MLKL S358D expression led to cell death, which could be blocked by the MLKL inhibitor necrosulfonamide (NSA) (Figures 4a and b).^21^ In contrast, ponatinib did not prevent cell death driven by MLKL S358D expression, strongly suggesting that this protein is not a direct drug target. Next, we assessed the effect of ponatinib on RIPK1 and RIPK3 by monitoring their phosphorylation status upon necroptosis induction (Figure 4c). Time-dependent phosphorylation of RIPK1, RIPK3 and MLKL in TSZ-treated HT-29 cells was blocked by ponatinib. Indeed, competitive binding assays demonstrated that ponatinib could directly bind RIPK1 *in vitro* with high affinity (*K*_d_: 37 nM) (Figure 4d). The impact of ponatinib and other BCR-ABL inhibitors on the catalytic activity of recombinant RIPK1 and RIPK3 was assessed in kinase assays (Figure 4e and Supplementary Figure 4b). Ponatinib strongly blocked phosphorylation of the generic substrate myelin basic protein (MBP), indicating inhibitory activity on both kinases. In agreement with the protective effect seen in FADD-deficient Jurkat cells at high drug concentration (Figure 2a), dasatinib also interfered with RIPK3 activity at the concentration tested (10 *μ*M) (Figure 4e). Ponatinib blocked recombinant RIPK3 with an IC_50_ of 0.64 *μ*M in this kinase assay (Figure 4f). We further monitored RIPK3 autophosphorylation (Supplementary Figure 4c), which was efficiently blocked by ponatinib, whereas the RIPK1 inhibitor Nec-1s showed no effect. Considering the different IC_50_ values observed for cellular viability and these *in vitro* kinase assays, we set out to verify whether endogenous RIPK3 is targeted by ponatinib in cells at the concentration (0.5 *μ*M) used throughout this study. To this end, we performed cellular thermal shift assays (CETSA), monitoring protein stabilization induced by drug binding.^51, 55^ Indeed, ponatinib treatment led to a shift in the RIPK3 thermostability curve, indicative of target engagement (Figures 4g and h). Moreover, in contrast to Nec-1, ponatinib was capable of preventing phosphorylation of MLKL induced by RIPK3 or MLKL overexpression (Figure 4i and Supplementary Figure 4d). In line with these results, ponatinib efficiently blocked binding of RIPK3 to MLKL (Figure 4j). These data demonstrate the ability of ponatinib to directly target RIPK1 and RIPK3, the two key mediators of necroptosis signaling. Besides pazopanib directly binding (*K*_d_=260 nM) ^50^ and inhibiting RIPK1 kinase activity (Supplementary Figure 4b), it did not block MLKL S358D-driven necroptosis (Supplementary Figure 5a) and only moderately affected RIPK3 activity in recombinant kinase assays (Supplementary Figure 5b). Similar to Nec-1, pazopanib blocked TSZ-induced phosphorylation of MLKL, while it had only a minor effect when MLKL phosphorylation was triggered by RIPK3 or MLKL overexpression (Figure 4i and Supplementary Figure 4d). Furthermore, pazopanib did not interfere with the binding of RIPK3 to MLKL (Figure 4j). Taken together these data point to RIPK1 as the main mediator of necroptosis inhibition by pazopanib.

## Discussion

In this study we performed a cellular screen with FDA-approved drugs to identify necroptosis inhibitors. While tool compounds blocking necrotic cell death by targeting RIPK1,^28, 35^ RIPK3^12^ and MLKL^21, 34^ have been developed, no necroptosis inhibitors are in clinical use to date. Dabrafenib has been recently proposed as a possible candidate.^56^ Our screen identified two kinase inhibitors, ponatinib and pazopanib, blocking necroptosis in human cells at submicromolar EC_50_ concentrations. Both drugs inhibited necroptotic signaling triggered by various cell death receptors, whereas they did not interfere with apoptosis. Regarding the mode of action underlying the necroptosis inhibition by pazopanib, our results suggested that the effect is not mediated through its established targets. Indeed, vandetanib, which has a largely overlapping target spectrum, comprising VEGFR-2, KIT and PDGFR, did not protect from necroptotic cell death.^42, 47^ Altogether the data point to RIPK1 as the main functional target mediating the protective effect of pazopanib. Pazopanib is used for treatment of advanced renal cell carcinoma^40^ and advanced soft tissue sarcoma^41^ at a daily dose of 800 mg, resulting in plasma concentrations between 20 and 40 *μ*M.^57, 58^ In our cellular systems, pazopanib conferred full necroptosis protection at 1–5 *μ*M concentration, suggesting a large therapeutic window for potential clinical application. Ponatinib was developed to treat patients having acquired the BCR-ABL T315I mutation, which confers resistance to all other clinically approved ABL1 tyrosine kinase inhibitors.^38^ In our experimental settings, ponatinib was more potent in inhibiting necroptosis than the widely used RIPK1 inhibitor Nec-1s. To understand the molecular basis for the inhibitory action of ponatinib, we investigated its target profile by performing drug affinity purifications. Interestingly, in contrast to other clinical BCR-ABL inhibitors,^45, 46^ ponatinib targets the key components of the necroptosis signaling pathway: RIPK1, RIPK3 and MLKL, as well as TAK1, TAB1 and TAB2. Our data support that ponatinib directly binds and inhibits both RIPK1 and RIPK3. Despite the relatively high IC_50_ value observed in *in vitro* RIPK3 kinase assays, effective target engagement and inhibition at the concentration used in cellular assays were demonstrated by thermal protein stabilization as well as loss of MLKL binding and phosphorylation. Thus ponatinib is, to our knowledge, the first necroptosis inhibitor capable of concurrently targeting both RIPK1 and RIPK3. The relative contribution of single kinase inhibition to the cellular protection cannot be readily assessed. However, this property of ponatinib could be advantageous in protecting cells from a broader range of stimuli, including those acting independently of RIPK1 or RIPK1 kinase activity.^12, 59^ In addition, ponatinib bound TAK1 with high affinity (*K*_d_=0.6 nM) (Supplementary Figure 6a). Besides its well established function in NF-*κ*B activation and prevention of cell death, TAK1 has recently been proposed to have a role in RIPK1/RIPK3-dependent necroptosis by taking part in a positive feed forward loop.^52, 53^ A specific TAK1 inhibitor was not able to confer protection to necroptosis in our system, indicating that necroptosis inhibition by ponatinib could not be mediated solely by targeting TAK1 (Supplementary Figures 6b and c). In terms of drug concentration, the effective dose needed for necroptosis inhibition lies within plasma levels (120–140 nM) obtainable in patients treated with the recommended oral dose of 45 mg ponatinib given once daily.^39^ While blocking RIPK3 protects cells from a broader range of necroptosis-inducing stimuli than RIPK1 inhibition, recent studies have raised concern about the therapeutic potential of targeting RIPK3 kinase activity.^60, 61^ Selective small-molecule RIPK3 inhibitors, as well as a kinase-dead RIPK3 D161N mutant protein, have been shown to promote caspase-8-dependent apoptosis.^60, 61^ While the RIPK3 D161N mutation is embryonically lethal,^61^ kinase-dead *Rip3*^K51A/K51A^ mice develop normally.^60^ This indicates that RIPK3 kinase activity *per se* is not required for viability and suggests that additional conformational changes are required to trigger apoptosis.^60^ Thus, development of therapeutically valuable RIPK3 inhibitors can be envisaged. Ponatinib and pazopanib have been developed and approved as anti-cancer treatment, and their safety profiles have been evaluated in this context, with both drugs reported to cause severe side effects.^62, 63^ In this regard, chemical proteomic profiling of ponatinib identified several novel targets in addition to RIPK1 and RIPK3. The definition of the cellular target spectrum might be useful in gaining a better understanding of the molecular mechanisms underlying the adverse effects reported in leukemia patients undergoing long-term drug treatment.^62, 64^ Necroptosis inhibitors hold most promise for treatment of clinical conditions in which necroptotic cell death can be anticipated as, for example, in ischemia-reperfusion damage following transplantation or vessel occlusion.^65^ These situations would require only single or short-term inhibitor treatment. Therefore potential side effects triggered by ponatinib or pazopanib in such acute settings might differ from those described for long-term anti-cancer treatment and would require cautious evaluation. The identification of two FDA-approved drugs as new inhibitors of necroptosis, together with elucidation of their mechanism of action, warrants a series of careful studies in animal models covering a large variety of necroptosis-associated pathologies. These studies will clarify the potential for necroptosis-related clinical application of these drugs which, given their potency in cellular assays and favorable pharmacological properties, could otherwise serve as basis for optimization in the development of drug-like necroptosis inhibitors.

## Materials and Methods

### Cell culture and reagents

HEK293T, 3T3-SA and L929 were obtained from ATCC (Manassas, VA, USA) and ECACC (Salisbury, UK). Jurkat E6.1 were kindly provided by W Ellmeier (Vienna), FADD-deficient Jurkat I2.1 and HT-29 by P Schneider (Lausanne). Cells were cultured in DMEM (Sigma-Aldrich, St. Louis, MO, USA), MEM (Gibco, Grand Island, NY, USA) or RPMI medium (Sigma-Aldrich) supplemented with 10% (v/v) FBS (Gibco) and antibiotics (100 U/ml penicillin and 100 mg/ml streptomycin) (Sigma-Aldrich). The reagents used were as follows: recombinant human TNF-*α* (300-01A, Peprotech, Rocky Hill, NJ, USA), recombinant murine TNF-*α* (315-01A, Peprotech), necrostatin-1 (N9037, Sigma-Aldrich), RIPK1 Inhibitor II 7-Cl-O-Nec-1 (Nec-1s) (504297, Merck Millipore, Billerica, MA, USA), ponatinib (S1490, Selleck Chemicals, Houston, TX, USA), pazopanib (P-6706, LC Laboratories, Woburn, MA, USA), imatinib (I-5577, LC Laboratories), nilotinib (S1033, Selleck Chemicals), dasatinib (S1021, Selleck Chemicals), vandetanib (S1046, Selleck Chemicals), SMAC mimetic Birinapant (S7015, Selleck Chemicals), z-VAD-FMK (AG-CP3-0002, Adipogen, San Diego, CA, USA), FasLigand (ALX-522-020-C005, Enzo, Farmingdale, NY, USA), recombinant human TRAIL (310-04, Peprotech), BMS-345541 (B9935, Sigma-Aldrich), c-ponatinib (WuXi AppTec, Shanghai, China), doxycycline (D9891, Sigma-Aldrich), NSA (480073, Merck Millipore), TAK1 inhibitor NP-009245 (AnalytiCon Discovery GmbH, Potsdam, Germany), propidium iodide (P4170, Sigma-Aldrich), recombinant RIPK3 (R09-10G, Signalchem, Richmond, BC, Canada), recombinant RIPK1 (R07-10G, Signalchem), [*γ*-32 P]ATP (SRP-30, Hartmann Analytic GmbH, Braunschweig, Germany) and MBP (ab792, Abcam, Cambridge, UK). Bosutinib was a kind gift from Oridis Biomed (Graz, Austria).

### Antibodies

Antibodies used were p-IkBa (Ser32/36) (9246S, Cell Signaling, Danvers, MA, USA), actin (AAN01-A, Cytoskeleton, Denver, CO, USA), IkBa (SC-371, Santa Cruz, Dallas, TX, USA), tubulin (ab7291 Abcam), phospho-MLKL (Ser385) (ab187091, Abcam), RIPK1 (610458, BD Biosciences, Franklin Lakes, NJ, USA), RIPK3 (12107, Cell Signaling), HA (SC-805, Santa Cruz) and HA-7 (H6533, Sigma-Aldrich). The secondary antibodies used were goat anti-mouse HRP (115-035-003, Jackson ImmunoResearch, West Grove, PA, USA), goat anti-rabbit HRP (111-035-003, Jackson ImmunoResearch), Alexa Fluor 680 goat anti-mouse (A-21057, Molecular probes, Grand Island, NY, USA) and IRDye 800 donkey anti-rabbit (611-732-127, Rockland, Limerick, PA, USA).

### Plasmids

RIPK3^66^ and MLKL (PCR-amplified from KBM7 cDNA) were subcloned into vector pDONR221 using Gateway technology (Invitrogen, Grand Island, NY, USA). MLKL S358D mutant was generated with the QuikChange Lightning Site-Directed Mutagenesis Kit (Agilent Technologies, Santa Clara, CA, USA) using primers MLKL_S358D_fw (3′-GGAA AACACAGACTGACATGAGTTTGGGAACT-5′) and MLKL_S358D_rv (3′-AGTTCCCA AACTCATGTCAGTCTGTGTTTTCC-5′). Following sequence verification, cDNAs were transferred into the Gateway-compatible expression vectors TSHgwICPB or TgwSHICPB with N-(RIPK3) or C-terminal (MLKL, MLKL S358D) Strep-HA tag, respectively.

### Generation of HT-29 cell lines with inducible overexpression

HT-29 cells were retrovirally transduced with MSCV-RIEP vector (pMSCV-rtTA3-IRES-EcoR-PGK-PuroR)^67^ to generate ecotropic receptor and rtTA3 co-expressing cell lines. In brief, HEK293T cells were transiently transfected with pGAG-POL, pVSV-G, pADVANTAGE and MSCV-RIEP. After 24 h the medium was replaced with fresh medium. Forty-eight hours later the virus-containing supernatant was harvested, filtered (0.45 *μ*m), supplemented with 8 *μ*g/ml protamine sulfate (Sigma-Aldrich) and added to 40–60% confluent HT-29 cells. Twenty-four hours after infection, the medium was replaced with fresh medium. Another 24 h later, the medium was supplemented with 2 *μ*g/ml puromycin (Sigma-Aldrich) to select for infected cells. rtTA3-expressing HT-29 cells were similarly transduced with retrovirus produced in HEK293T cells using the respective target gene-encoding inducible expression vector and pGAG-POL, pADVANTAGE and pEcoEnv. Blasticidin (25 *μ*g/ml; Invivogen, San Diego, CA, USA) was used for selection of infected cells. Target gene expression was induced by adding 1–2 *μ*g/ml doxycycline.

### Immunoblotting

Whole cell extracts were prepared using Nonidet-40 lysis buffer (1% NP-40, 50 mM HEPES pH 7.4, 250 mM NaCl, 5 mM EDTA, one tablet of EDTA-free protease inhibitor (Roche Diagnostics, Indianapolis, IN, USA) per 50 ml, 10 mM NaF and 1 mM Na_3_VO_4_) for 10 min on ice. Lysates were cleared by centrifugation in a microcentrifuge (13 000 r.p.m., 10 min, 4 °C). Proteins were quantified with BCA (Pierce, Grand Island, NY, USA). Cell lysates were resolved by SDS-PAGE and transferred to nitrocellulose membranes Protran BA 85 (GE Healthcare, Little Chalfont, UK). The membranes were immunoblotted with the indicated antibodies. The bound antibodies were visualized with horseradish peroxidase-conjugated antibodies against rabbit or mouse IgG using the ECL Western blotting system (GE Healthcare) or Odyssey Infrared Imager (LI-COR, Lincoln, NE, USA).

### Immunoprecipitation

Immunoprecipitations were performed as previously described.^68^ In brief, the lysates were precleared (30 min, 4 °C) on Sepharose6 beads (Sigma-Aldrich) and subsequently incubated (3 h, 4 °C) with monoclonal anti-HA agarose antibody (Sigma-Aldrich). Beads were recovered by centrifugation and washed three times with lysis buffer before analysis by SDS-PAGE and immunoblotting.

### Viability assays

Cells were seeded in 12-, 24- or 96-well plates at proper cell density. For necroptosis or apoptosis assays, cells were incubated with the indicated compound combinations at concentrations stated for 14–24 h. Smac mimetic and z-VAD-FMK were added 30–60 min before treatment with TNF-*α*, FasL or TRAIL. Cell viability was determined using CellTiter Glo Luminescent Cell Viability Assay (Promega, Fitchburg, WI, USA) according to the manufacturer's instructions. Luminescence was recorded with a SpectraMax M5Multimode plate reader (Molecular Devices, Sunnyvale, CA, USA). For flow cytometry-based determination of viability, cells were harvested, collected by centrifugation, washed once and resuspended in staining buffer (1 × PBS, 10% FBS). Cells were incubated with 0.5 *μ*g/ml PI for 10 min at room temperature (RT) in the dark. Flow cytometric analyses were performed on an LSR Fortessa (BD Biosciences) and analyzed with FlowJo software version 7.6.3 (Tree Star Inc, Ashland, OR, USA). Cell size was evaluated by forward scatter. PI-negative normal-sized cells were considered living and data were normalized to values of untreated controls. For detection of apoptosis, cells were resuspended in Annexin V Binding Buffer (BioLegend, San Diego, CA, USA) and stained with 0.5 *μ*g/ml PI and Alexa Fluor 647 Annexin V (BioLegend) according to manufacturer's instructions.

### Drug screen

The drug screen was performed in 384-well plates at a final compound concentration of 1.5 *μ*M and 0.5 *μ*M measured in duplicates. The compounds were transferred into drug plates by acoustic droplet ejection using an Echo 520 liquid handler (LABCYTE, Sunnyvale, CA, USA). Necroptosis was induced in FADD-deficient Jurkat cells by addition of 10 ng/ml TNF-*α* immediately before seeding onto drug plates at a density of 1x10^4^ cells/well. After 16 h incubation, cell viability was determined using CellTiter Glo Luminescent Cell Viability Assay according to manufacturer's instructions. Data analysis was performed by calculating a percentage of control to normalize for variability across different plates. The signal for the negative control DMSO wells was set to 0%, whereas the wells containing the positive control Nec-1 were put to 100% for each plate individually. Screen hits were defined as compounds (i) whose normalized signal was at least 3 S.D. away from the DMSO control, and (ii) that gave >20% rescue compared with the DMSO controls.

### Chemical proteomics

Drug-affinity matrices were prepared as described previously.^51^ In brief, 25 nmol of c-ponatinib were immobilized on 50 *μ*l NHS-activated Sepharose 4 Fast Flow beads (GE Healthcare Bio-Sciences AB, Uppsala, Sweden). 10 mg whole cell lysate was used as protein input per replicate. Affinity chromatography and elution were performed in duplicate. After elution, the enriched proteins were reduced with dithiothreitol (DTT) and cysteine residues were alkylated by incubation with iodoacetamide. The samples were digested with modified porcine trypsin (Promega). Five percent of the digested eluates were purified and concentrated by C18 reversed-phase material. Subsequently, samples were analyzed in duplicates by gel-free one-dimensional LCMS.

### MS data analysis

Precursor and MS/MS peaks were extracted from the RAW files and saved in the MGF format using msconvert tool (ProteoWizard Library v2.1.2708, www.proteowizard.sourceforge.net) for subsequent protein identification. An initial database search was performed with broader mass tolerance and conservative score threshold to re-calibrate the mass lists for optimal final protein identification. For the initial protein database search, Mascot (www.matrixscience.com, v2.3.02) was used. Error tolerances on the precursor and fragment ions were ±10 p.p.m. and ±0.6 Da, respectively, and the database search limited to fully-tryptic peptides with maximum one missed cleavage, carbamidomethyl cysteine and methionine oxidation set as fixed and variable modifications, respectively. The Mascot peptide ion score threshold was set to 30, and at least three peptide identifications per protein were required. Searches were performed against the human UniProtKB/SwissProt database (www.uniprot.org release 2013.01) including all protein isoforms. The initial peptide identifications were used to deduce independent linear transformations for precursor and fragment masses that would minimize the mean square deviation of measured masses from theoretical. Re-calibrated mass list files were searched against the same human protein database by a combination of Mascot and Phenyx (GeneBio SA, Geneva, Switzerland; version 2.5.14) search engines using narrower mass tolerances (±4 p.p.m. and ±0.3 Da). One missed tryptic cleavage site was allowed. Carbamidomethyl cysteine was set as a fixed modification and oxidized methionine was set as a variable modification. To validate the proteins, Mascot and Phenyx output files were processed by internally developed parsers. Proteins with ≥2 unique peptides above a score T1, or with a single peptide above a score T2 were selected as unambiguous identifications. Additional peptides for these validated proteins with score >T3 were also accepted. For Mascot searches, the following thresholds were used: T1=14, T2=40 and T3=10; Phenyx thresholds were set to 4.2, 4.75 and 3.5, respectively (*P*-value<10^−3^). The validated proteins retrieved by the two algorithms were merged, any spectral conflicts discarded and grouped according to shared peptides. A false discovery rate of <1% for protein identifications and <0.1% for peptides (including the ones exported with lower scores) was determined by applying the same procedure against a database of reversed protein sequences. The statistical significance of protein enrichment in c-ponatinib assay *versus* free ponatinib competition assay was calculated using the modification of the Decontaminator method.^69^ In addition to Mascot protein scores that were proposed as an estimate of protein abundance by the original algorithm, the modified version also included Phenyx protein scores and spectral counts. The algorithm first estimated three measure-specific *P*-values for each putative interaction, and, in contrast to,^69^ *P*-value calculation utilized the quantiles of the measurements rather than the original data. The Fisher's method was used to combine the three resulting *P*-values into a single *P*-value for drug–protein interaction specificity. We have also correlated interaction *P*-values with the magnitude of competition effect represented by the fold reduction of spectral counts on free compound competition. Fold reduction was computed as the ratio of median spectral counts observed in experiments with and without competition. In each condition, four spectral counts were available for the median (two biological replicates and two technical for each).

### Competition binding assays

Competition binding assays were performed by DiscoveRx (Fremont, CA, USA) according to the following protocol: kinase-tagged T7 phage strains were prepared in an *Escherichia coli* host derived from the BL21 strain. *E. coli* were grown to log phase and infected with T7 phage and incubated with shaking at 32 °C until lysis. The lysates were centrifuged and filtered to remove cell debris. The remaining kinases were produced in HEK293 cells and subsequently tagged with DNA for qPCR detection. Streptavidin-coated magnetic beads were treated with biotinylated small-molecule ligands for 30 min at RT to generate affinity resins for kinase assays. The liganded beads were blocked with excess biotin and washed with blocking buffer (SeaBlock (Pierce), 1% BSA, 0.05% Tween-20 and 1 mM DTT) to remove unbound ligand and to reduce nonspecific binding. Binding reactions were assembled by combining kinases, liganded affinity beads and test compounds in 1x binding buffer (20% SeaBlock, 0.17x PBS, 0.05% Tween-20 and 6 mM DTT). All reactions were performed in polystyrene 96-well plates in a final volume of 0.135 ml. The assay plates were incubated at RT with shaking for 1 h and the affinity beads were washed with wash buffer (1x PBS and 0.05% Tween-20). The beads were then resuspended in elution buffer (1x PBS, 0.05% Tween-20 and 0.5 *μ*M nonbiotinylated affinity ligand) and incubated at RT with shaking for 30 min. The kinase concentration in the eluates was measured by qPCR. For kinase-binding constant determination, an 11-point 3-fold serial dilution of each test compound was prepared in 100% DMSO at 100x final test concentration and subsequently diluted to 1x in the assay (final DMSO concentration=1%). Most *K*_d_s were determined using a compound top concentration=30 000 nM. If the initial *K*_d_ determined was <0.5 nM (the lowest concentration tested), the measurement was repeated with a serial dilution starting at a lower top concentration. A *K*_d_ value reported as 40 000 nM indicates that the *K*_d_ was determined to be >30 000 nM. Binding constants (*K*_d_) were calculated with a standard dose–response curve using the Hill equation:

Response=background+{(signal−background)/[1+(*K*_d_ Hill Slope/Dose Hill Slope)]}.

The Hill Slope was set to −1. Curves were fitted using a non-linear least square fit with the Levenberg–Marquardt algorithm.

### Recombinant kinase assays

Recombinant RIPK1 or RIPK3 protein was incubated with the substrate MBP in kinase assay buffer (40 mM Tris-HCl pH 7.5, 10 mM MgCl_2_ and 1 mM DTT) in presence of 50 *μ*M ATP, 5 *μ*Ci [*γ*-32 P]ATP and the indicated inhibitors in a total volume of 20 *μ*l. Reactions were incubated at RT for 20 min before termination by addition of 12.5 *μ*l guanidinium chloride (7.5 *μ*M). The terminated reactions were spotted onto a SAM2 Biotin Capture membrane (Promega) and further processed according to the manufacturer's instructions. Kinase activity was measured in a Tri-Carb 2810TR liquid scintillation analyzer (Perkin Elmer, Waltham, MA, USA). Data were normalized to a DMSO-treated control sample. For autoradiography, the reactions were stopped by addition of 5 *μ*l 4x Laemmli buffer and incubated for 5 min at 95 °C. Samples were resolved by SDS-PAGE followed by gel drying for 2 h at 80 °C in a Model 583 Gel Dryer (Bio-Rad, Hercules, CA, USA). Visualization was performed using a BAS-IP MS 2025 imaging plate (Fuji Photo Film Co., Ltd., Tokyo, Japan) and a Typhoon TRIO Variable Mode Imager (GE Healthcare).

### Cellular thermal shift assay (CETSA)

Drug target engagement in cells, causing stabilization of the respective protein, was analyzed essentially as described previously.^55^ Briefly, FADD-deficient Jurkat cells were seeded into 12-well plates at a density of 1x10^6^ cells/ml and treated for 3 h with cell media containing 0.5 *μ*M ponatinib or 0.05% DMSO. After treatment, cells were collected by centrifugation and resuspended in 1x PBS. The cell suspension was aliquoted into PCR tubes and heated for 3 min at 42, 46, 50, 54, 58 or 62 °C. Subsequently, cells were lysed by three consecutive freeze–thaw cycles using liquid nitrogen. The soluble fraction was separated from precipitated proteins by centrifugation at 13000 r.p.m. and 4 °C for 20 min. The supernatant, containing the soluble proteins, was transferred to a fresh tube and analyzed by immunoblotting.

### Microscopy

Microscopy images were taken at 10x with a Leica DFC310 FX on a Leica DM IL LED (Leica Microsystems, Wetzlar, Germany).

## Abbreviations

CETSA: cellular thermal shift assay
EC_50_: half maximal effective concentration
FADD: Fas-associated protein with death domain
FasL: Fas ligand
FDA: Food and Drug Administration
IC_50_: half maximal inhibitory concentration
LCMS-MS: liquid chromatography tandem mass spectrometry
MBP: myelin basic protein
MLKL: mixed lineage kinase domain-like protein
Nec-1: necrostatin-1
NSA: necrosulfonamide
PDGFR*β*: platelet-derived growth factor receptor *β*
RIPK: receptor-interacting serine/threonine-protein kinase
TAB1: TGF-beta-activated kinase 1 and MAP3K7-binding protein 1
TAK1: transforming growth factor-*β*-activated kinase 1
TLR: Toll-like receptor
TNF*-α*: tumor necrosis factor-alpha
TRAIL: TNF-related apoptosis-inducing ligand
VEGFR: vascular endothelial growth factor receptor
