# Supplementary figures and images for: A cellular screen identifies ponatinib and pazopanib as inhibitors of necroptosis
Source: Cell Death Dis. 2015 May 21;6(5):e1767–. doi: 10.1038/cddis.2015.130 (PMC4669708; doi:10.1038/cddis.2015.130)

Supplementary Figure 1

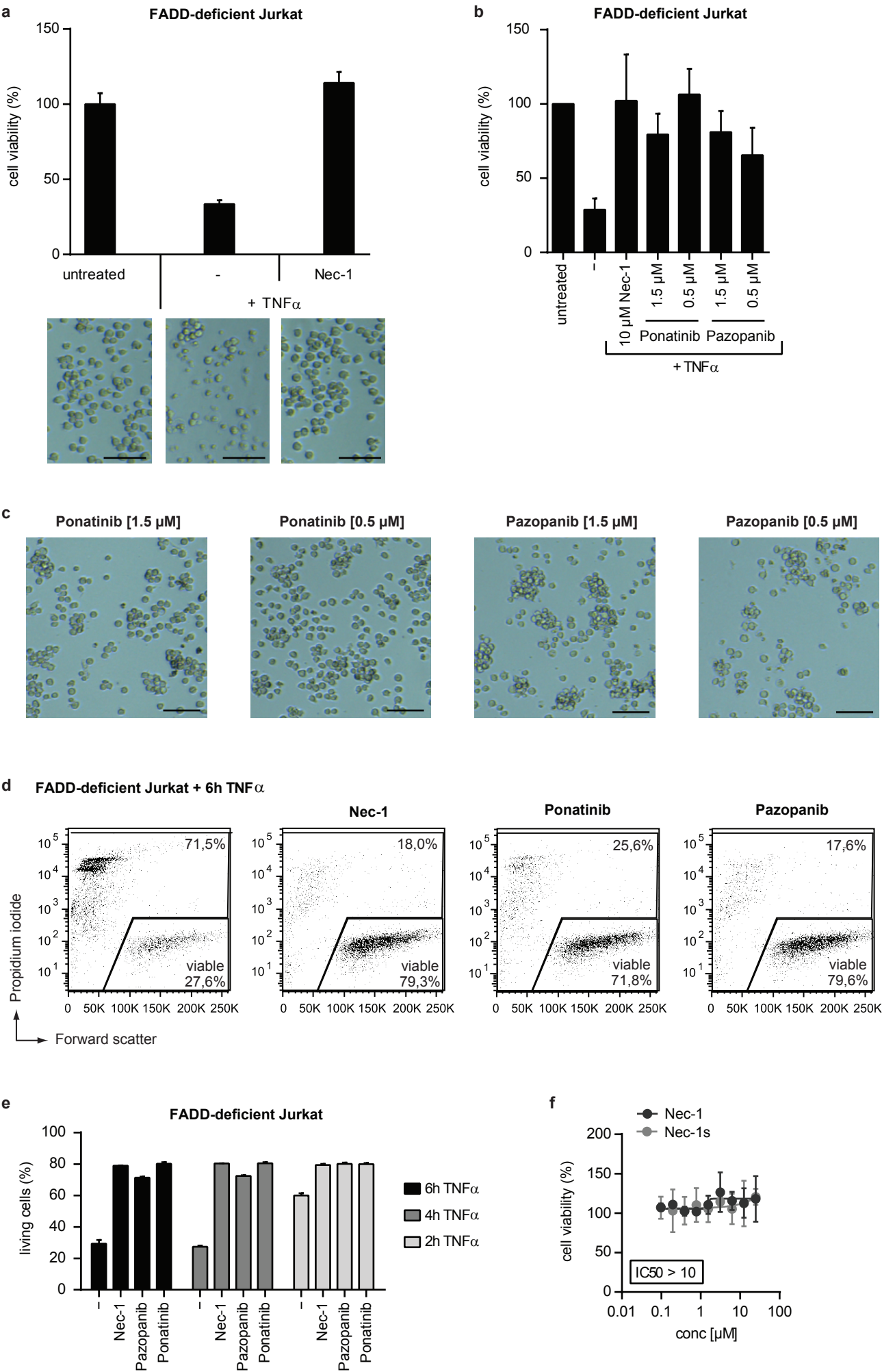

Supplement: Supplementary Figure 1 [file cddis2015130x1.pdf]

Supplementary Figure 2

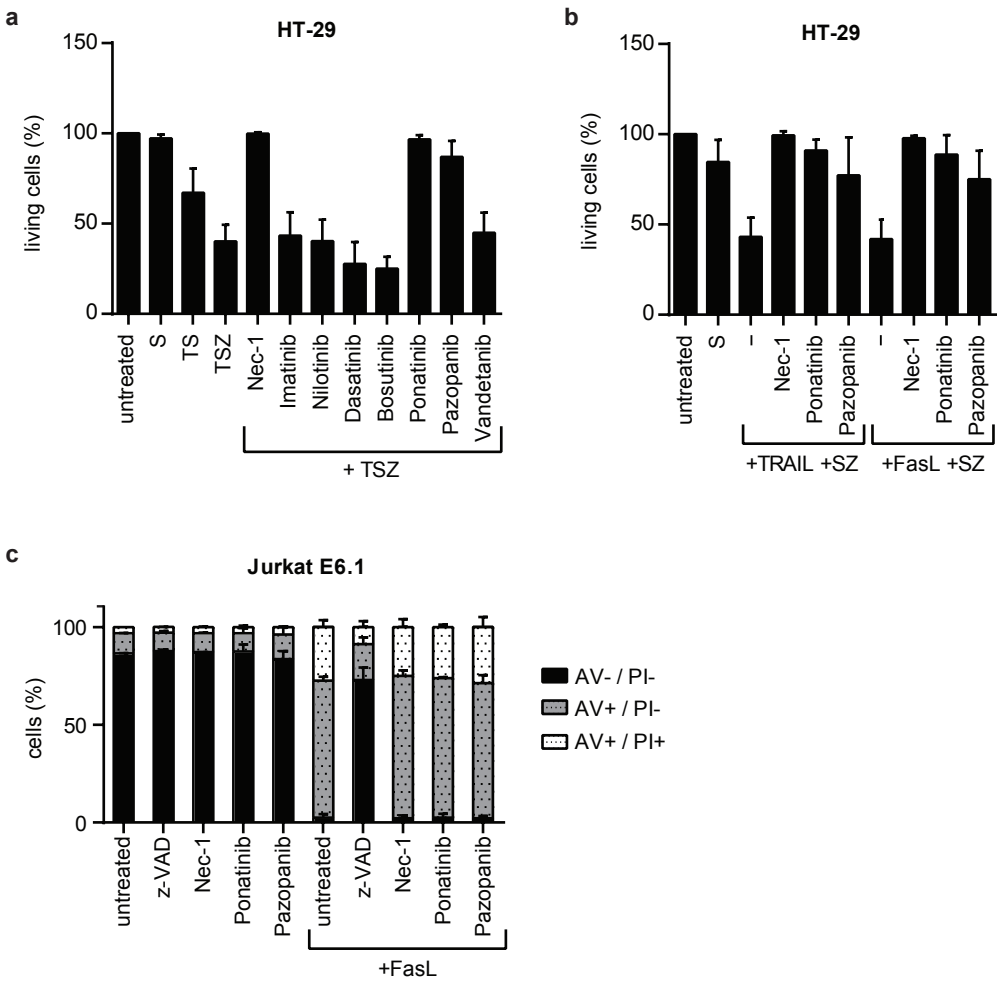

Supplement: Supplementary Figure 2 [file cddis2015130x2.pdf]

Supplementary Figure 3

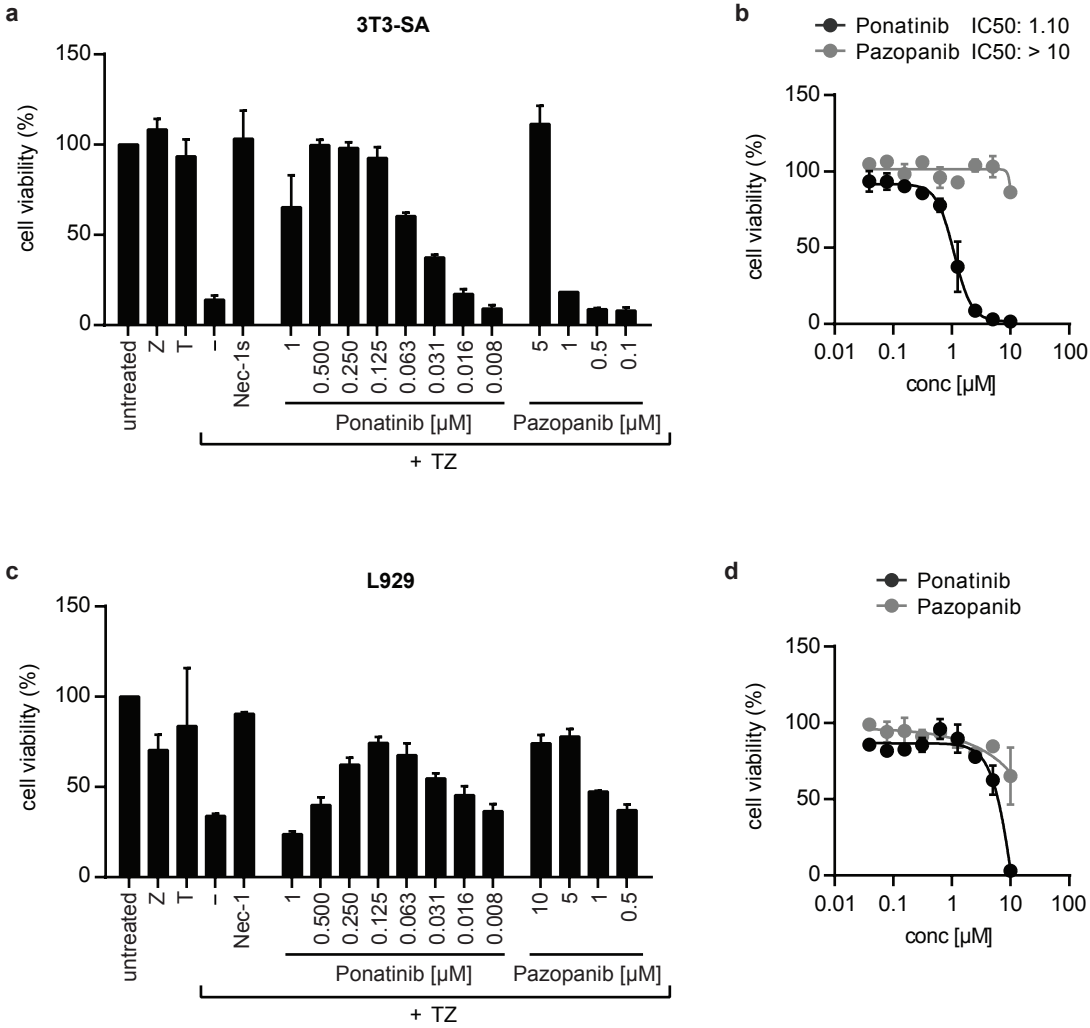

Supplement: Supplementary Figure 3 [file cddis2015130x3.pdf]

Supplementary Figure 4

a HT-29 MLKL S358D

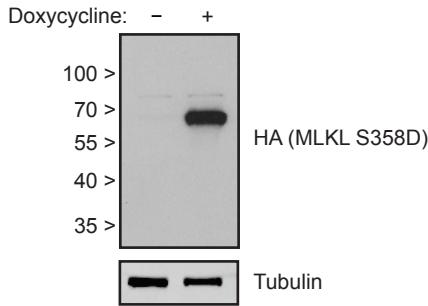

b RIPK1

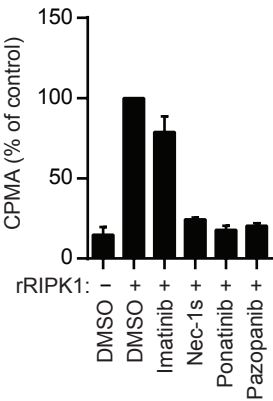

c RIPK3

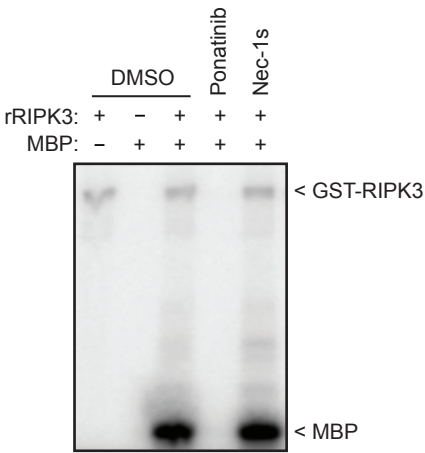

d HT-29 MLKL

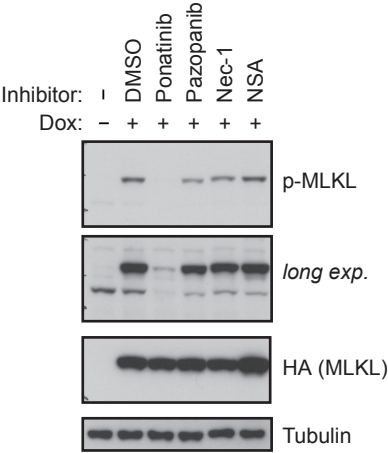

Supplement: Supplementary Figure 4 [file cddis2015130x4.pdf]

Supplementary Figure 5

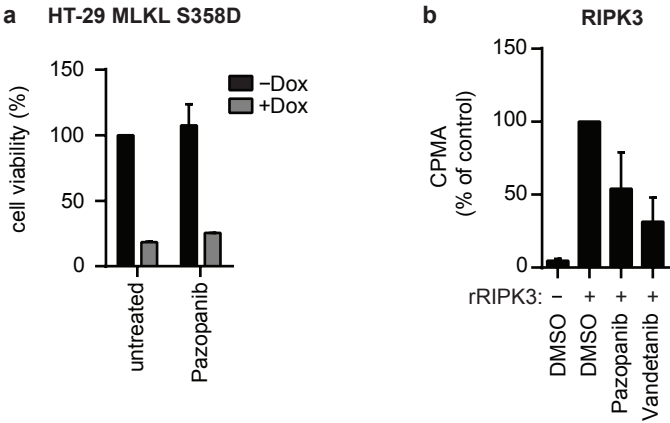

Supplement: Supplementary Figure 5 [file cddis2015130x5.pdf]

Supplementary Figure 6

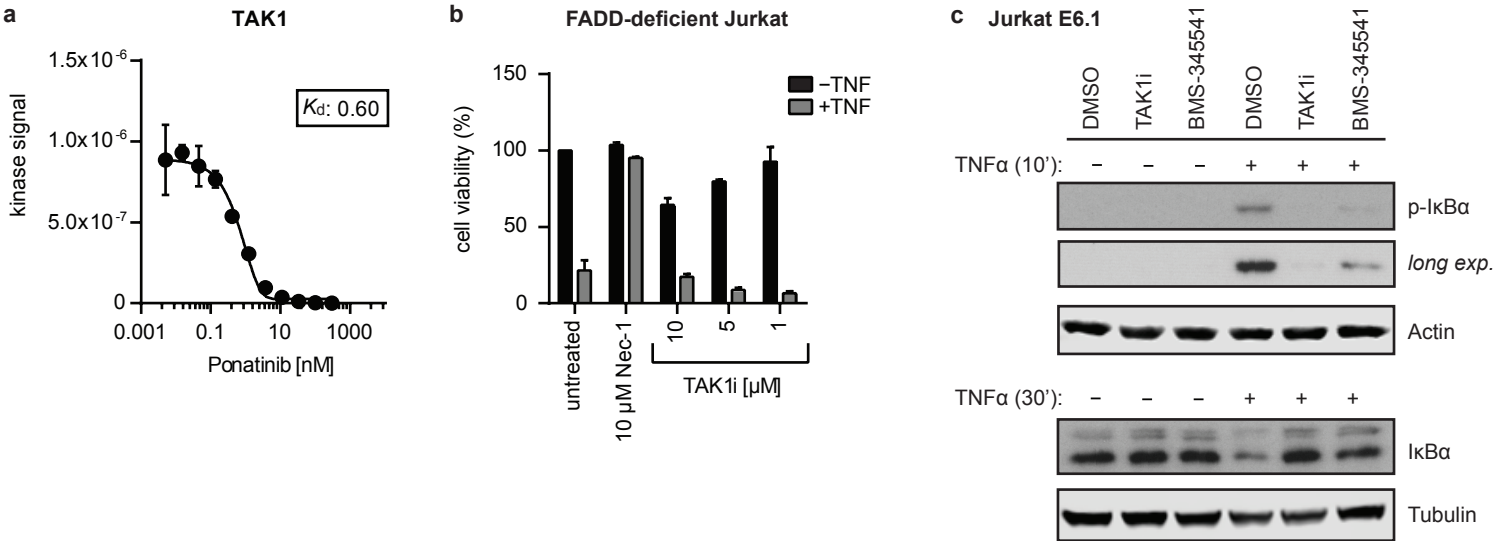

Supplement: Supplementary Figure 6 [file cddis2015130x6.pdf]
