# Supplementary material for: A cellular screen identifies ponatinib and pazopanib as inhibitors of necroptosis
Source: Cell Death Dis. 2015 May 21;6(5):e1767–. doi: 10.1038/cddis.2015.130 (PMC4669708; doi:10.1038/cddis.2015.130)
Supplement: Supplementary Table 1 [file cddis2015130x7.pdf]

Supplementary Table 1: Average (Av.) and total spectral counts (SC) for proteins identified by LC-MS/MS analysis in c-ponatinib pulldowns performed without or with competing (comp.) free ponatinib. Kinases are highlighted in **bold** and kinase targets shared by other BCR-ABL inhibitors (45, 46) are marked with *. Proteins involved in necroptosis signaling are highlighted in red.

Protein label	Full protein name	Av. SC	Total SC	Av. SC comp.	Total SC comp.	p-value	
**MAP4K1**	Mitogen-activated protein kinase kinase kinase kinase 1	46.75	187	2.5	10	6.34E-10	*
**ZAK**	Mitogen-activated protein kinase kinase kinase MLT	65.25	261	0	0	2.12E-09	*
**LCK**	Tyrosine-protein kinase Lck	98.5	394	0.75	3	2.91E-09	*
**CSK**	Tyrosine-protein kinase CSK	52.25	209	4.5	18	1.28E-08	*
**PTK2B**	Protein-tyrosine kinase 2-beta	32.5	130	0	0	1.37E-08	*
**MAP3K7**	Mitogen-activated protein kinase kinase kinase 7	19.5	78	2.25	9	4.72E-08	
YWHAB	14-3-3 protein beta/alpha	7	28	1	4	5.18E-08	
**TNIK**	TRAF2 and NCK-interacting protein kinase	26.75	107	0	0	5.6E-08	*
**JAK1**	Tyrosine-protein kinase JAK1	21.5	86	0	0	5.81E-08	
**MAPKAPK2**	MAP kinase-activated protein kinase 2	23.5	94	0	0	6.16E-08	
YWHAE	14-3-3 protein epsilon	13	52	1	4	8.52E-08	
**CDK16**	Cyclin-dependent kinase 16	10.75	43	1.25	5	1.28E-07	
**ABL2**	Abelson tyrosine-protein kinase 2	17.25	69	0	0	1.37E-07	*
**RIPK3**	Receptor-interacting serine/threonine-protein kinase 3	17	68	0	0	1.56E-07	
UNC119	Protein unc-119 homolog A	20.5	82	0	0	2.01E-07	
**ABL1**	Abl1 human spliceform 1a (myristoylated)	13.75	55	0	0	2.23E-07	*
**TAOK3**	Serine/threonine-protein kinase TAO3	13	52	2	8	2.52E-07	*
**MAPK14**	Mitogen-activated protein kinase 14	79.25	317	7.25	29	2.7E-07	*
**IRAK1**	Interleukin-1 receptor-associated kinase 1	24.25	97	4	16	3.54E-07	
**TYK2**	Non-receptor tyrosine-protein kinase TYK2	14	56	0	0	3.65E-07	*
**MAPKAPK3**	MAP kinase-activated protein kinase 3	10.5	42	0	0	5.53E-07	
TAB1	TGF-beta-activated kinase 1 and MAP3K7-binding protein 1	38.25	153	6.75	27	7.74E-07	
**MAP3K1**	Mitogen-activated protein kinase kinase kinase 1	9	36	0	0	1.01E-06	*
**BLK**	Tyrosine-protein kinase Blk	9.5	38	0	0	1.12E-06	*
YWHAQ	14-3-3 protein theta	9	36	1.25	5	1.38E-06	
PLEKHF2	Pleckstrin homology domain-containing family F member 2	8.25	33	0	0	1.41E-06	
YWHAZ	14-3-3 protein zeta/delta	10.5	42	2.5	10	1.61E-06	
YWHAH	14-3-3 protein eta	6	24	0.25	1	1.98E-06	
**IRAK4**	Interleukin-1 receptor-associated kinase 4	8.5	34	2.5	10	1.99E-06	*
**HCK**	Tyrosine-protein kinase HCK	6	24	0	0	4.48E-06	*
**MAP4K4**	Mitogen-activated protein kinase kinase kinase kinase 4	54.5	218	10.75	43	4.64E-06	*
UNC119B	Protein unc-119 homolog B	6	24	0	0	5.89E-06	
**STK10**	Serine/threonine-protein kinase 10	5.5	22	0	0	1.23E-05	*
HSPA5	78 kDa glucose-regulated protein	7	28	2.75	11	1.38E-05	
**MAPK12**	Mitogen-activated protein kinase 12	10.75	43	3.75	15	1.4E-05	
**TESK2**	Dual specificity testis-specific protein kinase 2	5	20	0	0	1.57E-05	*
**MLKL**	Mixed lineage kinase domain-like protein	5	20	0	0	1.82E-05	
**CDK19**	Cyclin-dependent kinase 19	7.5	30	0	0	2.77E-05	
**GAK**	Cyclin-G-associated kinase	22.75	91	10.25	41	3.19E-05	*
YWHAG	14-3-3 protein gamma	11.25	45	3.25	13	4.76E-05	
**MINK1**	Misshapen-like kinase 1	4	16	0	0	6.42E-05	*
CCT4	T-complex protein 1 subunit delta	3.5	14	0	0	2.55E-04	
**CDK8**	Cyclin-dependent kinase 8	3.75	15	0	0	3.19E-04	
HSPA4	Heat shock 70 kDa protein 4	5.5	22	1.75	7	4.09E-04	
**MELK**	Maternal embryonic leucine zipper kinase	5	20	1	4	4.66E-04	
HSPA9	Stress-70 protein, mitochondrial	3.25	13	0	0	9.74E-04	
TCP1	T-complex protein 1 subunit alpha	5	20	1.5	6	1.23E-03	
**MAPK11**	Mitogen-activated protein kinase 11	3	12	0	0	1.40E-03	*
TAB2	TGF-beta-activated kinase 1 and MAP3K7-binding protein 2	2.5	10	0	0	2.99E-03	
CCT2	T-complex protein 1 subunit beta	3.5	14	0	0	5.50E-03	
TUBA1B	Tubulin alpha-1B chain	18.5	74	10.25	41	5.73E-03	
**MAP4K2**	Mitogen-activated protein kinase kinase kinase kinase 2	2.5	10	0	0	7.21E-03	
**JAK2**	Tyrosine-protein kinase JAK2	2.25	9	0	0	1.35E-02	
**CDK5**	Cyclin-dependent kinase 5	7.25	29	2.75	11	1.52E-02	
PAG1	Phosphoprotein associated with glycosphingolipid-enriched microdomains 1	2	8	0	0	1.54E-02	
TPM4	Tropomyosin alpha-4 chain	3.5	14	0.25	1	1.80E-02	
HSPA8	Heat shock cognate 71 kDa protein	29.25	117	17.25	69	2.04E-02	
**RIPK1**	Receptor-interacting serine/threonine-protein kinase 1	1.5	6	0	0	2.25E-02	
NAP1L1	Nucleosome assembly protein 1-like 1	2	8	1.25	5	2.29E-02	
**RAF1**	RAF proto-oncogene serine/threonine-protein kinase	1.25	5	0	0	4.76E-02	
